# A comprehensive psychometrics of cognitive ability measures: Reliability, practice effects, and the stability of latent factor structures across retesting

**DOI:** 10.3758/s13428-025-02897-8

**Published:** 2026-01-29

**Authors:** Matthew K. Robison, Stephen Campbell, Lauren D. Garner, Ciara Sibley, Joseph Coyne

**Affiliations:** 1 University of Notre Dame, Notre Dame, IN 46556 USA; 2 U.S. Naval Research Laboratory, Washington, DC 20375 USA

**Keywords:** Attention control, Working memory, Long-term memory, Short-term memory, Spatial ability, Fluid intelligence, Reliability

## Abstract

The present study examined individual differences in 24 measures of cognitive ability in a sample of young adults (*N* = 255). Each measure was completed twice, separated by a period of 2 weeks, to assess test–retest reliability and retesting (i.e., practice) effects. Latent variable modeling was used to assess the convergent and discriminant validity of the measures, as they were selected to measure seven different cognitive constructs (attention control, processing speed, working memory, primary memory, secondary memory, fluid intelligence, and spatial ability). The measures showed adequate to high intrasession and intersession reliability. Construct-level estimates were highly reliable, and the measurement structure was invariant across the two testing occasions. In several instances, correlations among latent variables warranted further testing to ensure adequate discriminability. Finally, latent state-trait modeling indicated that the majority of systematic variance in cognitive measures is due to latent traits, rather than state-specific or task-specific factors. We discuss the practical and theoretical implications of these findings.

In addition to controlled between- and within-person experiments, the evaluation of individual differences in a psychological phenomenon is one of the two primary means by which psychologists test theories (Cronbach, 1957; Underwood, 1975). Within cognitive psychology, we use the correlational structure of observations to develop and test theories regarding the fundamental properties of human cognition. The psychometric properties of the tools we use to measure cognitive processes and abilities are thus the bedrock upon which any differential investigation rests. In the present study, we conducted a comprehensive psychometric analysis of cognitive ability measures. Specifically, we examined task- and construct-level reliability, retest (i.e., practice) effects, latent structural stability across repeated testing, and the relative variance in measures attributable to task-specific factors, state-specific factors, and stable task-general traits. We included measures of attention control, processing speed, working memory, primary (i.e., short-term) memory, secondary (i.e., long-term) memory, fluid intelligence, and spatial abilities. In doing so, we hoped to examine the extent to which measures of these cognitive constructs (a) demonstrate adequate intrasession and test–retest reliability, (b) change with practice, (c) form coherent latent factors, (d) maintain that factor structure across repeated measurements, and (e) are influenced by task- and state-specific factors.

The primary impetus for the current study was recent debate regarding the psychometric properties of attention control as a cognitive construct. The debate started when differential psychologists repeatedly found poor psychometric properties (e.g., low reliability, minimal intercorrelation) for putative measures of attention control (Draheim et al., 2019; Enkavi et al., 2019; Hedge et al., 2018; Rey-Mermet, 2024; Rey-Mermet et al., 2018, 2019; Rouder & Haaf, 2019; Rouder et al., 2023; von Bastian et al., 2020; Whitehead et al., 2019, 2020). The crux of the problem is that many attention tasks that work quite well for experimental purposes (e.g., Stroop, flanker, Simon) do not demonstrate adequate intrasession or intersession reliability, nor do they correlate with each other to an extent that would be expected if they measured a common cognitive construct. This was considered problematic for theories that posit attention control as a centrally important construct, giving rise to individual differences in important cognitive functions (e.g., reading comprehension, multitasking, intelligence, learning new languages; Burgoyne et al., 2023; Engle, 2002, 2018; McVay & Kane, 2012; Redick et al., 2016; Robison et al., 2024a, 2024b; Unsworth & Spillers, 2010; Unsworth et al., 2014). In response, new measures of attention control have been developed (Burgoyne et al., 2023), older measures have been modified or repurposed for individual-differences investigations (Draheim et al., 2021, 2023), or alternatives to button presses, such as mouse-tracking, have been used as alternatives (Unsworth & Miller, 2024). Doing so has improved the reliability of the tools used to measure attention control and demonstrated theorized latent-level coherence among those measures such that an “attention control” construct can be implicated.

Despite the improvements in the internal consistency and test–retest reliability of individual measurements, views still differ regarding the validity of the tools—the extent to which they measure the construct they are designed (or assumed) to measure. Again, with a specific critique lobbed at attention control, some have argued that the major—and perhaps the only—important source of shared variance among classic measures of attention control is processing speed (Löffler et al., 2024; Schubert et al., 2017), whereas others have argued that individual differences in attention control measure more than just processing speed (Burgoyne et al., 2023; Mashburn et al., 2024; Robinson & Steyvers, 2023; Tsukahara et al., 2025), and that processing speed and attention control, while related, account for separable sources of variance in higher-order cognition. Further, a recent meta-analysis and re-analysis of several decades’ worth of latent variable analyses of attention control indicate the presence of a coherent latent variable for attention control (Unsworth et al., 2024).

Although the focus of recent cognitive psychometric research has been on attention control, we took a broader approach to determining the psychometrics of individual measures and latent constructs. We did so because our theories often do not consider just attention control, but structural relations among the many facets of human cognition—attention, processing speed, short- and long-term memory, and reasoning. Before we can start to test our theories using a differential approach, the psychometric properties of individual measures and their putative underlying constructs need to be firmly established. The present study was a step toward establishing such properties, or potentially, a lack thereof.

Here we examined five specific aspects of cognitive measures. First, we examined intrasession and test–retest reliability. For most tasks, intrasession reliability was computed using a split-half procedure where we separately estimated a task’s dependent measure on two halves of trials (typically odd trials and even trials), correlated the measures across the halves, and then applied the Spearman–Brown split-half correction to the correlation. For test–retest reliability, we correlated the first and second attempt at each measure and also computed the intraclass correlation coefficient. We considered reliability to be acceptable if estimates were ≥.70. Establishing adequate reliability is a critical step for most psychological measurements, but it is especially true for differential approaches. If a measure’s reliability is low, then most of its variance is simply noise. Theoretically, then, reliability places an upper bound on the extent to which a measure can correlate with any other measure. However, if there is systematic interindividual variance in the measure, then we can be relatively certain that the measure is capturing something stable within people, which is our goal.

Second, we examined practice effects on each measure. Practice effects can arise for a variety of reasons (see Hausknecht et al., 2007; Scharfen et al., 2018a, 2018b, 2018c, for meta-analyses). For example, people may develop a task-specific strategy for completing the task. Or they may develop more automaticity with response mappings, allowing for faster responses where reaction times are the dependent measure. In these cases, people will show a positive practice effect. That is, performance scores will improve from a first to second attempt. However, it is also possible that participants will show negative practice effects. For example, in memory tasks, a buildup of proactive interference may carry over from one attempt to the next, or participants may lose motivation and therefore provide less effort on a second attempt. To the extent that a measure is affected by motivation or effort, scores will drop. While practice effects are sometimes unavoidable, they need not be problematic for an individual-differences design. As long as the relative rank-ordering of individuals does not change, the reliability and correlational structure of the data may remain intact. But this is a notion that will be tested here.

Third, we examined the latent factor structure of the measures using confirmatory factor analysis. We chose measures that have been used previously to tap into each of six cognitive constructs. Using factor analysis, we test the degree to which our chosen measures tap into common constructs, as hypothesized. Here, we are testing for both convergent and discriminant validity (Campbell & Fiske, 1959). That is, measures of a common construct should correlate with each other and should load onto a common factor in a latent variable analysis (i.e., convergent validity). To demonstrate discriminant validity, we need to demonstrate that measures of different constructs (e.g., short-term and long-term memory) do indeed show a factor structure reflecting independence of the constructs. Of course, cognitive measures tend to correlate with one another (i.e., the positive manifold; Spearman, 1904). Therefore, we do not expect any of the constructs to be orthogonal (i.e., correlated at or near 0). But if the correlation between two factors approaches 1, we may be overparameterizing the model. In those cases, we will need to verify that a model which specifies two highly correlated factors does not fit better than a more parsimonious model with just one factor. In a sense, we are trying to avoid both *jingle* and *jangle* fallacies (Kelley, 1927). The *jingle* fallacy is the assumption that two measures reflect the same construct when in reality they measure two different constructs (i.e., a lack of convergent validity); the *jangle* fallacy is labeling two constructs differently when in reality they represent the same underlying construct (i.e., a lack of discriminant validity).

Fourth, we examined the stability of the latent factor structure by applying the same confirmatory factor model to the first and second attempts. By using measurement invariance tests, which force model parameters to be equal when fit to different datasets, we can evaluate whether the model parameters significantly differ from one dataset to another. If the parameters do indeed differ, fixing the parameters to be equal will worsen model fit, indicating that there is measurement *variance*—a significant difference in the magnitude of factor loadings, the magnitude of interfactor correlations, or both—between datasets. But if fixing model parameters to be equal does *not* change the model fit, then we can be assured we have measurement *invariance—*stability in the factor structure.

Finally, we used latent state-trait modeling to partition variance into individual measures. Much like any psychological measurement, any measure of cognitive ability/performance will have at least four sources of variance: (a) trait-level variance, (b) task-specific variance, (c) state-specific variance, and (d) measurement error. To the extent that the cognitive measures tap into task-general, stable individual differences in cognitive abilities, the interindividual variance and covariance among measures will be driven by trait factors. If instead the covariance is due to daily and/or momentary variations in states like alertness/fatigue, motivation, or stress, the covariance will be driven by state-specific factors. If participants are using strategies or mnemonics to complete the tasks, rather than any task-general ability per se, then the covariance will be driven by task-specific factors. The final source of variance, unattributable to trait, state, or task specificity, is measurement error. Using latent state-trait modeling allowed us to parse the variance in all 48 measures (24 tasks, each given twice) into these four components. While often applied to psychological factors like mood, personality, and psychopathology (Steyer et al., 1992; 1999), latent state-trait modeling has been applied to cognitive data as well. For example, Danner et al. (2011) examined fluid intelligence, dynamic decision-making, and implicit learning within a sample of working-age adults. For the measures of fluid intelligence, they found high reliability (i.e., low measurement error), high trait-level variance, and low task-specific variance. For the measures of dynamic decision-making, they found high reliability and about equal trait- and task-specific variability for one task, but low reliability for another. Measures of implicit learning had low reliability, and all systematic variability was due to trait-level factors. As another example, Hermes and Stelling (2016) examined five measures of cognitive ability within a sample of pilots applying for a position with an airline. The battery comprised measures of mental arithmetic, auditory memory, visual memory, visual perceptual speed, and selective attention. Each measure was given twice, separated by an average of 214 days, but with considerable variability (range = [13–841 days]). Hermes and Stelling estimated the state-specific proportion of variance to be 5%, whereas the trait/task-level variance was 71%, with the remaining variance being due to measurement error. Similarly, using a different approach, Schmidt et al. (2003) estimated the reliability of one cognitive ability test (Wonderlic) to be overestimated by about 5%, assigning this variance to be occasion-specific. Finally, Faßbender et al. (2023) recently applied latent state-trait models to measures of inhibitory control (e.g., antisaccade, Eriksen flanker, go/no-go) and found that the majority of variance in the measures was due to trait-level factors, not state-specific or task-specific variance.

Given the sheer number of cognitive facets under investigation here, it was of interest to us whether variance in measures would be differentially attributable to trait-level, state-specific, or task-specific variance depending on the construct each task was intended to measure. The results showed that the majority of variance in tasks was driven by trait-level variability and measurement error, followed by task specificity, with very little variance attributable to state specificity.

## Present study

We administered a comprehensive battery of computerized cognitive measures to a large sample of healthy young adults (*N* = 255). With the exception of attention control, which we measured using six different tasks, we used three tasks of each construct so we could conduct latent variable analysis on each of the seven constructs of interest (24 total tasks). The battery was split over 2 days of testing separated by 1 week, then repeated in the subsequent 2 weeks (i.e., one session per week for 4 weeks). This approach allowed us to test several psychometric properties of the measures. To be clear, much prior work has been conducted to assess the psychometrics of one or several of the constructs of interest here. Extensive work has been undertaken to examine the reliability of individual measures or constructs, many of which are included in our battery (Açıkgül et al., 2023; Burgoyne et al., 2023; Dai et al., 2019; Foster et al., 2015; Hutton & Ettinger, 2006; Redick et al., 2012; Xu et al., 2018), and the presence of retesting effects on these and similar measures has been widely explored as well (Hausknecht et al., 2007; Scharfen et al., 2018a, 2018b, 2018c). Thus, we do not contend that the psychometrics of these measures have never been evaluated. But we believe our approach advances beyond this work in several ways. The breadth and diversity of tasks used in the battery enables a robust assessment of cognitive abilities. This broad assessment allows us to examine the psychometrics (e.g., instrasession reliability, test–retest reliability, practice effects) from a relatively atheoretical perspective, while also addressing important theoretical issues in the psychometrics of cognition. Specifically, the use of latent variable modeling allows us to test specific questions regarding the measurement properties of cognitive tasks and constructs (e.g., convergent and discriminant validity). To preview the results, we will use the covariance structures to test specific theoretical issues regarding attention control and processing speed.

The present study has bearings on at least three areas of inquiry. Most directly, the findings will address the ongoing debate regarding the psychometrics of attention control and other constructs in the field of differential cognitive psychology. The results will also be of interest to developmental psychologists who may use these or similar cognitive measures to assess the trajectory of cognition across childhood, into adolescence and adulthood, and with aging, as poor psychometrics significantly hamper the interpretability of developmental differences (or lack thereof) in cognition. Further, the results will be of interest to clinicians who use cognitive assessments to evaluate individuals for the presence of clinically significant symptomologies. If the measures are not reliable or lack validity, their use as a clinical tool is questionable. Finally, interventions (e.g., pharmaceuticals, fitness programs, individual therapies) are often used to improve cognitive outcomes for specific populations, as either a direct or indirect effect of the intervention. Again, the efficacy of any given intervention must be weighed against the effects of simple practice. And perhaps more importantly, poor psychometrics can make the evaluation of an intervention’s efficacy almost impossible. Next, the results bear on the use of cognitive testing as a selection tool in educational and workplace settings. It is not uncommon for people to take assessments more than once, either because people are required to take the same assessments multiple times, or because students and job-seekers prepare for the tests ahead of time. Therefore, it is worth knowing whether practice with tasks fundamentally changes their measurement properties. For example, measures may be noisier at first as people learn the task’s instructions, response mappings, and requirements. Conversely, measures may become even noisier with repeated testing as people introduce their own idiosyncratic strategies, acquiesce to a minimal effort level, or learn to “game” the measures via hacks or shortcuts. Thus, the use of repeated testing allows for the assessment not only of the stability of cognitive measures across time, but also the degree to which first attempts at a task measure the same underlying source(s) of variance as second attempts. Finally, latent state-trait modeling will allow us to capture whether the covariance among measures is driven by state-specific factors, like fatigue, stress, and motivation, task-specific factors like strategies and mnemonics, or trait-level factors.

## Method

We report how we determined our sample size, all measures, all manipulations, and all exclusions, where appropriate.

### Participants and procedure

A sample of 272 participants, all students at the University of Texas at Arlington, completed at least one part of the study. After exclusions and dropouts, the final sample included 255 participants, 249 of whom completed all four sessions (*M*_age_ = 19.02 years, *SD*_age_ = 1.82, age range 17–34, 77% female/women, 22% male/men, 1% nonbinary; 43% White, 23% Asian, 23% Black or African American, 2% Native American, 35% Hispanic or Latino; 11% other race^1^; 71% native English speakers). On the basis of simulations for precise estimation of correlations (Schönbrodt & Perugini, 2013), we set a minimum target sample size of 200 participants, but we aimed to collect as many participants as possible over a 2-year period. Limitations on available funding and the relocation of the lab to a different university also necessitated the stoppage of data collection. Most of the data were collected during the fall 2022, spring 2023, fall 2023, and spring 2024 academic semesters. However, some participants completed the study during summer 2023. The study involved four separate 2.5-h sessions, completed on the same day of the week and time on consecutive weeks. In the event of a missed appointment or the need to reschedule, participants were allowed to make up a session on a different date and time. Participants were paid in cash, $40 each for sessions 1 through 3. Upon completing session 4, they were paid $80, with $40 given for completing the session and $40 as a bonus for completing all four sessions. Thus, total maximum compensation was $200. The experimental protocol was approved by the institutional review board of the University of Texas at Arlington.

Prior to completing any tasks, participants read and signed an informed consent document. Then, they completed a brief demographic questionnaire. The entire cognitive battery was split over sessions 1 and 2 and repeated on sessions 3 and 4. Table 1 lists the cognitive factors of interest and their associated definitions. Table 2 lists the tasks that were completed in the order in which they were administered. All participants completed the tasks in the same order. We decided to use a fixed order to (1) reduce the administrative burden on the experimenter to create different random orders for each participant for each session and (2) reduce the chances of introducing experimenter error (e.g., accidentally skipping tasks) by randomizing task sequencing.^2^ Participants completed the tasks in an individual run room in the dark. There were three sessions per day, starting at 9 AM, 12 PM, and 3 PM. Participants completed the tasks at their own pace. A research assistant initiated each task, answered any questions participants had, and noted any issues (e.g., crashes, freezes, errors, etc.). Participants were seated at a desk with a desktop computer, monitor, backlit keyboard, mouse, and hands-on-throttle-and-stick (HOTAS) device. The HOTAS was used only for a tracking task which participants completed three times in sessions 2 and 4. The results of this task will not be reported here. Eye-tracking data were collected during all tasks, so participants sat with their head position fixed in a chinrest for every task. Participants were seated such that their head position was ~ 60 cm from the monitor. The eye-tracking data will not be analyzed or reported here.

**Table 1:** Cognitive constructs of interest with associated definitions

Construct	Definition
Attention control	Maintenance of task goals for fast and accurate responding, especially in situations dealing with uncertainty and the overriding of automatic response tendencies
Processing speed	Quick and accurate perception of stimuli for translation of perception into an appropriate action
Working memory	Maintenance, conversion, and updating of goal-relevant mental representations
Primary memory	Short-term storage of goal-relevant representations for immediate access
Secondary memory	Encoding of goal-relevant representations into long-term storage for delayed access and retrieval
Fluid intelligence	Finding solutions to novel and abstract problems through inductive reasoning
Spatial ability	Using geometric structures to mentally rotate objects and determine spatial positioning

At the end of session 4, participants completed a set of questionnaires including a video game experience survey, the Big Five Inventory (John et al., 2008), the Perceptions of Academic Stress Scale (Bedewy & Gabriel, 2015), the Center for Epidemiologic Studies–Depression scale (Radloff, 1977), the Spielberger State-Trait Anxiety Inventory (Marteau & Bekker, 1992), and a media multitasking questionnaire (Ophir et al., 2009). Participants were also asked for their consent to release their academic records to the researchers. This consent could be withheld while still completing the remainder of the study. Finally, participants wore a Fitbit Charge 5 fitness band for the 3-week interval between sessions 1 and 4 to record their sleep and physical activity. None of these measures will be analyzed here (Table 2).

**Table 2:** Tasks completed and their order of administration

Sessions 1 and 3	Sessions 2 and 4
Operation span	Immediate free recall
Color change detection	Choice reaction time
Raven	Terrain orientation task
Antisaccade	Tracking task (1)
Symmetry span	Cued recall
Orientation change detection	Letter comparison
Number series	Spatial apperception task
Psychomotor vigilance	Tracking task (2)
Reading span	Recognition memory
Letter change detection	Digit comparison
Letter sets	Flanker^2^
SART	Stroop^2^
	Simon^2^
	Tracking task (3)
	Paper folding

### Tasks

#### Attention control

##### Antisaccade (Hutchison, 2007; Kane et al., 2001)

Each trial started with three fixation crosses (+ + +) centered on the screen for 2 s. Then, a centered cue appeared (***) for either 2 or 3 s. A flashing cue (=) then appeared on either the right or left size of the screen for 300 ms. Next, a target letter (O or Q) appeared on the opposite side of the screen as the cue for 100 ms, followed by a backward mask (##) until a participant made a response. Participants were to press the key (O or Q) associated with the target letter. There were eight practice trials at a slowed pace, followed by 16 practice trials at the normal experimental pace, then 60 experimental trials. The dependent variable was proportion correct.

##### Psychomotor vigilance (PVT; Dinges & Powell, 1985; Wilkinson & Houghton, 1982)

Each trial started with a 2-s fixation screen (+ + + + +) in black text centered against a gray background. Then, a row of underlined zeros (0.000) appeared in the same location. After an interval ranging randomly and uniformly from 2 to 8 s, the timer began counting forward. The participant’s task was to stop the timer as quickly as possible by pressing the spacebar. When they did so, their reaction time (e.g., 0.347) was shown to them for 2 s, followed by a brief intertrial interval. There were five practice trials followed by 60 experimental trials. The dependent variable was the mean of a participant’s slowest quintile of trials.

##### Sustained Attention to Response Task (Robertson et al., 1997)

Each trial presented a single digit (1–9) for 250 ms followed by a 1,500-ms mask (#). Participants were instructed to press the spacebar any time they saw a digit other than 3. When they saw a 3, they were to withhold their response. There were 27 practice trials followed by 360 experimental trials. Each digit was equally likely; thus there were 320 “go” trials and 40 “no-go” trials. The dependent variable was the standard deviation of reaction times to “go” trials.

##### Stroop^2^ (Burgoyne et al., 2023)

On each trial, participants were presented with a word, RED or BLUE, at the center of the screen in either red or blue font. Below the word were two response options, also the words RED or BLUE in either red or blue font. The participants’ task was to use the font color of the stimulus word to find the response associated below. Thus, the stimulus could be congruent or incongruent, as could the response options. The locations of BLUE and RED response options were also randomly intermixed between the right and left. Participants made their responses using the mouse. After 30 s of practice trials, they were given 90 s to complete as many trials as possible. The dependent variable was the number of correct responses minus the number of incorrect responses in the 90-s period (average number of trials completed = 43 in Attempt 1, 49 in Attempt 2).

##### Flanker^2^ (Burgoyne et al., 2023)

On each trial, a row of directional arrows (e.g., < < < < <) appeared at the center of the screen, with two response options shown below it. The response options were similar stimuli. The participants’ task was to find the response option whose innermost arrow matched the direction of the stimuli’s outermost arrows. Both the stimuli and response options could show full arrow congruency (e.g., < < < < <) or incongruency (e.g., < < > < <). After 30 s of practice trials, participants were given 90 s to complete as many trials as possible. The dependent variable was the number of correct responses minus the number of incorrect responses in the 90-s period (average number of trials completed = 35 in Attempt 1, 40 in Attempt 2).

##### Simon^2^ (Burgoyne et al., 2023)

On each trial, a single arrow appeared on either the right or left side of the screen. Below that appeared two response options, the words RIGHT or LEFT. The location of the RIGHT and LEFT response options was randomized across trials, as was the direction and horizontal location of the stimulus. Participants’ task was to find the word that matched the direction of the arrow. After 30 s of practice trials, participants were given 90 s to complete as many trials as possible. The dependent variable was the number of correct responses minus the number of incorrect responses in the 90-s period (average number of trials completed = 63 in Attempt 1, 65 in Attempt 2).

#### Processing speed

##### Digit comparison (Salthouse & Babcock, 1991)

On each trial, two 3-, 5-, or 7-digit strings appeared on either side of a line (e.g., 58193____58193). Participants were to indicate whether the two strings were an exact match or not by pressing either the “F” or “J” key on a keyboard, respectively. Upon pressing a key, the stimulus disappeared and a new item appeared after a 500-ms delay. Matches occurred on 50% of trials. Non-matches were created by changing a single digit. Participants completed two 30-s blocks of trials. The dependent variable was the total number of correct responses in the time limit (average number of trials completed = 36 in Attempt 1, 38 in Attempt 2).

##### Letter comparison (Salthouse & Babcock, 1991)

On each trial, two 3-, 5-, or 7-letter strings appeared on either side of a line (e.g., HCBYP____KCBYP). Participants were to indicate whether the two strings were an exact match or not by pressing either the “F” or “J” key on a keyboard, respectively. Upon pressing a key, the stimulus disappeared and a new item appeared after a 500-ms delay. Matches occurred on 50% of trials. Non-matches were created by changing a single letter. Participants completed two 30-s blocks of trials. The dependent variable was the total number of correct responses in the time limit (average number of trials completed = 33 in Attempt 1, 35 in Attempt 2).

##### Choice reaction time

Each trial started by showing four blank horizontal placeholders spaced equally across the screen (e.g.,). Then, after a jittered time, sampled from a uniform distribution ranging from 1 to 2 s in 100-ms intervals, an asterisk appeared over one of the locations, and participants reported the location as quickly as possible by pressing the “D,” “F,” “J,” or “K” keys on the keyboard. There were eight practice trials followed by 50 experimental trials. The dependent variable was the median of response times (RTs) on accurate responses.

#### Working memory

##### Operation span (Unsworth et al., 2005)

Participants were required to remember a list of letters while they completed interleaved math problems. On each trial, a math problem appeared (e.g., [3 × 4] – 2 = 10?). When the participant had solved the problem, they clicked the mouse, then saw two response options: True or False. After responding, a single letter appeared. At the end of a list ranging in length from three to seven items, participants were required to recall the letters from the list in forward serial order. The dependent variable was the total number of letters reported in their correct serial position. There were two lists of each length (max score = 50).

##### Symmetry span (Unsworth et al., 2009)

Participants were required to remember sequences of spatial locations while making interleaved symmetry judgments. On each trial, a black and white pattern appeared, and participants determined whether the pattern was symmetrical about its *y*-axis. After making the symmetry judgment, a black and white 4 × 4 grid appeared, and one square within the grid flashed red. Then, at the end of a list ranging in length from two to five items, the participants’ task was to report the locations that flashed in the grid in forward serial order. The dependent variable was the number of items reported in their correct serial position. There were two lists of each length (max score = 28).

##### Reading span (Unsworth et al., 2009)

Participants were required to remember a list of letters while they evaluated the sensibility of sentences. On each trial, a sentence appeared (e.g., “The prosecutor’s dish was lost due to lack of evidence.”). When the participant had made a decision, they clicked the mouse, then saw two response options: True or False. The task was to determine whether the sentence made sense or not. After responding, a single letter appeared. At the end of a list ranging in length from three to seven items, participants were required to recall the letters from the list in forward serial order. The dependent variable was the total number of letters reported in their correct serial position. There were two lists of each length (max score = 50).

#### Primary memory

##### Color change detection (Luck & Vogel, 1997)

Each trial started with a 1-s fixation screen which showed a black cross against a gray background. Then, a set of six colored squares appeared on the screen for 250 ms. After a 4,000-ms blank delay screen, the squares reappeared in the same locations. One square had a black circle around it. The participants’ task was to indicate whether this square was the same color as its first presentation or a different color. Participants used the “F” and “J” keys to indicate same or different, respectively. Tested items changed color on 50% of trials. Non-tested items never changed. There were five practice trials followed by 60 experimental trials. The dependent variable was accuracy.

##### Orientation change detection (Luck & Vogel, 1997)

Each trial started with a 1-s fixation screen which showed a black cross against a gray background. Then, a set of six oriented bars appeared on the screen for 250 ms. After a 4,000-ms blank delay screen, the bars reappeared in the same locations. One square had a black circle around it. The participants’ task was to indicate whether this square was the same orientation, or a different orientation, than its first presentation. Participants used the “F” and “J” keys to indicate same or different, respectively. Tested items changed orientation on 50% of trials. Non-tested items never changed. There were five practice trials followed by 60 experimental trials. The dependent variable was accuracy.

##### Letter change detection (Robison & Brewer, 2020)

Each trial started with a 1-s fixation screen which showed a black cross against a gray background. Then, a set of six letters, sampled from the English consonants, appeared on the screen for 250 ms. After a 4,000-ms blank delay screen, the letters reappeared in the same locations. One square had a black circle around it. The participants’ task was to indicate whether this square was the same color as its first presentation or a different color. Participants used the “F” and “J” keys to indicate same or different, respectively. Tested items changed on 50% of trials. Non-tested items never changed. There were five practice trials followed by 60 experimental trials. The dependent variable was accuracy.

#### Secondary memory

##### Immediate free recall

Participants were given lists of 10 words to remember. Each word was presented individually in black text against a gray background for 3 s, followed by a 1-s mask screen (#####). All words were five letters in length. At the end of the encoding period, participants immediately began the recall phase. Participants recalled the words by typing them into a text box on the screen and pressing the “Enter” key to submit each response. Participants were allowed to recall the words in any order they wished. Responses were recorded as accurate if they came from the immediately preceding encoding list (i.e., previous list intrusions and repetitions were excluded from the count). There were 10 lists in total. All participants received the same word lists, but different lists were given on session 2 and session 4. The dependent variable was the average number of words recalled per list (max score = 10).

##### Cued recall

Participants were given lists of 10 word pairs to remember. All pairs comprised two 4-letter, unrelated words (e.g., FROG–CAPE). Each pair was presented for 3 s, followed by a 1-s mask (#### – ####). At the end of the encoding period, half of the pair was presented as a cue, and the participant was asked to recall the paired word (e.g., FROG – _____). Cue words were delivered in random order (i.e., the testing order did not match the encoding order). Participants had a maximum of 5 s to type in the target word. There were 10 lists in total. All participants received the same lists, but different lists were given on session 2 and session 4. The dependent variable was the average number of correctly recalled target words (max score = 10).

##### Word recognition

Participants were presented with a list of 20 words. Each word was presented for 3 s, followed by a 1-s mask (######). All words were six letters in length. At the end of the encoding period, participants were presented with a new, randomly shuffled list of 20 words, 10 of which had been on the encoding list, 10 of which were new words. The participants’ task was to determine whether each word was “old” or “new” using the “F” and “J” keys, respectively. Participants were given a maximum of 5 s to make a response; otherwise the response was marked as incorrect. There were five lists in total. All participants received the same word lists, but different lists were given on session 2 and session 4. The dependent variable was accuracy (max score = 1.0).

#### Fluid intelligence

##### Raven Advanced Progressive Matrices (Raven et al., 1962)

On each trial, participants saw a 3 × 3 grid of geometric patterns. The bottom-right piece of the grid was missing. Based on an implicit set of rules, participants were to select from a set of eight possible options the piece that best completed the grid. Participants completed the 18 odd-numbered items in session 1, and the 18 even-numbered items in session 3. There was a 10-min time limit to complete as many items as possible. The dependent variable was the total number of correct responses (max score = 18).

##### Number series (Thurstone, 1938)

Participants were given a set of numbers and asked to select from a set of five possible options which number best continued an implicit pattern. Participants were given 4.5 min to complete as many items as possible. There were 15 items in total. All participants received the same items in the same order, but different items were given at session 1 and session 3. The dependent variable was the total number of correct responses (max score = 15).

##### Letter sets (Ekstrom & Harman, 1976)

Participants were given five sets of four letters (e.g., ABAA CDCC XYXX HGHH) and asked to select the set that did not match the pattern present in the other three sets. Participants were given 5 min to complete as many items as possible. There were 15 items in total. All participants received the same items in the same order, but different items were given at session 1 and session 3. The dependent variable was the total number of correct responses (max score = 15).

#### Spatial ability

##### Terrain orientation task (Coyne et al., in press)

This task is a new spatial ability measure being evaluated by the U.S. Navy for pilot selection. The test is currently part of the Aviation Selection Test Battery (ASTB), and the modified version evaluated here uses the same type of images, time limits, and number of items as the official test. The test was designed to measure terrain association, the ability to identify objects on a map, and then use them to determine the direction of travel. The task presents participants with two images: (1) a reference map that is always oriented with north at the top, and (2) a map of the same area but oriented in a different direction. The second map is said to be from a vehicle with a downward-looking camera. The task is to determine what direction the vehicle is traveling. Participants were given 12 practice trails with feedback, including animation on incorrect trials, which showed how the terrain in map 2 changed as a vehicle changed its heading. After the 12 practice trials, participants completed 18 trials without feedback. The maps for each of the 18 trials were unique. Participants had 30 s to complete each trial. The dependent variable was the number of correct responses.

##### Spatial apperception test

This test has been used in aviation selection since World War II. Guilford (1947) listed it as the aerial orientation task in their report on classification tests. The version used in the current study was taken from an earlier version of the Navy’s Aviation Selection Test Battery (ASTB) and was last used for selection purposes in 2013. The paper items were scanned and presented on a computer to automate logging and ensure the time limit was enforced. The test shows a visualization that represents a view from an aircraft as the leftmost image, and then five images of aircraft flying over different terrain and at different attitudes (pitch, roll, and yaw). The participant had to determine which image of an aircraft flying would result in the visualization on the left. The test consisted of four practice items with feedback, followed by a timed portion in which participants could answer up to 35 items in 10 min. The dependent variable was the number of correct responses minus the number of incorrect responses within the time limit.

##### Paper folding (Ekstrom & Harman, 1976)

On each trial, the participant is shown a series of two to four sequences in which a piece of paper is folded. In the final sequence, a hole is punched through the paper. The participant must then select among five choices which image correctly represents the number and placement of holes in the paper when unfolded. The version of this task consisted of one practice item with feedback, followed by two timed sections in which participants had up to 3 min in each section to answer up to 10 items. The maximum number of items across both sections was 20. Participants had the option of skipping items. The dependent variable was the number of correct responses.

### Data analysis

The data were analyzed in R (R Core Team, 2024) using the *data.table* (Barrett et al., 2024), *tidyverse* (Wickham, 2016), *psych* (Revelle, 2024), *lavaan* (Rosseel, 2012), *RAMpath* (Zhang et al., 2023), *effectsize* (Ben-Shachar et al., 2020), *ggplot2* (Wickham, 2016), *bayestestR* (Makowski et al., 2019), *cowplot* (Wilke, 2024), and *papaja* (Aust & Barth, 2023) packages. For most tasks, intrasession reliability was estimated by splitting trials into odd and even trial numbers, calculating the dependent variable on each half, correlating the scores, and applying the Spearman–Brown split-half correction to the correlation. For the recognition memory task, reliability was computed as Cronbach’s α on accuracy on each list. For the complex span tasks, reliability was computed as Cronbach’s α on accuracy by set size. For all tasks, intersession reliability was computed as the correlation between a participant’s first and second attempts on each task and an intraclass correlation coefficient (ICC; two-way mixed effects, mean of two measurements, consistency; Koo & Li, 2016; Shrout & Fleiss, 1979). Practice effects were computed using a paired-samples comparison, subtracting performance on Attempt 1 from performance on Attempt 2. We report Cohen’s *d* with a 95% confidence interval around the effect size estimate. Unless otherwise noted, we used an α level of.05, two-tailed, to assess statistical significance. We adjusted the α level to correct for multiple comparisons when necessary.

For the latent variable models, we used *z*-scores for each task, calculated separately for each attempt. Fit was assessed using the χ^2^ test, the comparative fit index (CFI), the Tucker–Lewis index (TLI), root mean square error of approximation (RMSEA), and standardized root mean residual (SRMR). We considered model fit acceptable if CFI and TLI were ≥.90 and RMSEA and SRMR were ≤.08. While there are no definitive thresholds for fit indices, we used these cutoffs based on typical benchmarks within cognitive psychology. For all models, we report standardized factor loadings, residual variances, and interfactor correlations. When comparing nested models, we used a χ^2^ test with the associated change in degrees of freedom and Bayes factor (BF) comparison using the Bayesian information criterion for the nested models (Wagenmakers, 2007).

For the latent state-trait model, measures from the same task were specified to load onto 24 task factors. For each construct, the three task factors were then loaded onto trait factors. Measures taken during the same session were allowed to load onto four state factors. Two measures from the same task were specified to load equally onto their task factor and onto their associated state factors. Loadings onto trait factors were freely estimated. The correlations among task factors, state factors, and trait factors were set to zero. The trait factors were allowed to correlate. Upon fitting the model, the variances for the digit comparison and letter comparison task factors were negative, so these were instead set to be 0.

### Exclusions

Several participants withdrew from the study during or after session 1. These participants were not included in the analysis. We then used the following criteria to exclude problematic data: for the PVT task, we excluded RTs shorter than 200 ms and longer than 3,000 ms; for the SART, we excluded participants who made responses on fewer than 50% of “go” trials; for the choice RT task, we excluded trials with RTs shorter than 200 ms or longer than 5,000 ms and participants with less than 75% accuracy.^3^ For all measures, to ensure multivariate normality, we performed a multi-pass outlier exclusion by removing participants who fell more than ± 3 SDs outside the mean. This exclusion criterion was applied iteratively to each task until no more cases were removed. All other missing cases were due to computer crashes, freezes, or other errors.

## Results

Descriptive statistics for participants’ first and second attempts at each task are listed in Table 3. Zero-order correlations among the measures for first and second attempts are listed in Tables 4 and 5, respectively. On Attempt 1, most measures showed acceptable reliability estimates (>.70), with the only exception being symmetry span (α =.68). All measures also demonstrated normal distributions (|skew|< 1, |kurtosis|< 2). On Attempt 2, these results held, but reliabilities were now all above.70 and all measures continued to show normal distributions.

**Table 3:** Descriptive statistics for all measures separated by attempt

	Attempt 1						Attempt 2					
Measure	*N*	Mean	*SD*	Skew	Kurtosis	ρ	*N*	Mean	*SD*	Skew	Kurtosis	ρ
Antisaccade	248	0.69	0.14	–0.13	–0.97	.84	244	0.70	0.16	–0.03	–1.17	.89
Psychomotor vigilance	218	573.29	115.87	0.85	0.33	.86	223	659.28	184.72	1.01	0.46	.85
SART	223	142.33	70.26	0.92	0.34	.93	223	154.96	82.57	0.99	0.54	.97
Stroop^2^	241	29.91	15.24	–0.56	–0.27	.93	238	36.35	16.40	–0.66	–0.46	.92
Flanker^2^	242	23.53	14.22	–0.42	–0.48	.98	240	28.86	14.48	–0.59	–0.26	.98
Simon^2^	238	59.64	9.23	–0.37	–0.28	.92	233	60.93	9.85	–0.36	–0.23	.94
Choice reaction time	234	483.65	76.12	0.45	–0.35	.96	227	485.42	76.25	0.50	–0.13	.96
Letter comparison	231	28.06	4.45	0.14	0.05	.80	234	28.50	4.42	0.16	0.14	.82
Digit comparison	236	31.11	4.08	0.12	–0.15	.73	229	31.86	4.51	–0.05	0.26	.80
Operation span	244	36.31	9.29	–0.76	0.10	.78^α^	233	38.60	7.87	–0.73	0.06	.83^α^
Symmetry span	239	21.05	4.48	–0.51	–0.53	.68^α^	236	22.00	4.58	–0.73	0.00	.75^α^
Reading span	245	33.26	10.07	–0.67	–0.17	.87^α^	245	32.03	11.93	–0.73	–0.03	.87^α^
Color change detection	246	0.69	0.11	–0.59	0.36	.74	243	0.66	0.12	–0.14	–0.63	.76
Orientation change detection	248	0.65	0.12	0.03	–0.31	.77	244	0.62	0.12	0.33	–0.38	.75
Letter change detection	248	0.65	0.11	–0.17	–0.57	.72	245	0.64	0.13	0.17	–0.91	.79
Immediate free recall	233	5.05	1.39	0.17	–0.01	.87	230	5.19	1.76	0.22	–0.21	.92
Cued recall	239	0.49	0.28	–0.02	–1.32	.93^α^	241	0.48	0.30	0.06	–1.34	.94^α^
Word recognition	236	0.74	0.14	–0.17	–0.69	.92	235	0.69	0.15	–0.14	–0.95	.93
Raven	248	8.33	3.42	0.02	–0.15	.75	244	6.79	3.49	0.07	–0.81	.78
Number series	250	7.14	2.67	0.25	–0.19	.72	243	6.85	2.57	0.30	0.36	.72
Letter sets	241	6.65	2.51	–0.16	–0.13	.71	229	7.46	2.78	–0.19	–0.44	.75
Paper folding	243	10.25	4.10	–0.09	–0.66	.84	240	10.46	4.37	–0.28	–0.87	.87
Spatial apperception	244	–5.86	10.59	0.33	–0.26	.79	241	–4.24	12.11	0.46	–0.35	.83
Terrain orientation	232	5.16	3.76	0.69	–0.43	.77	237	5.98	4.52	0.52	–0.83	.85

*SD* standard deviation, *ρ* intrasession reliability estimate, *SART* Sustained Attention to Response Task. Unless otherwise noted, reliability was measured via splitting odd/even trial numbers, computing the dependent variable on each half, correlating the halves, and applying the Spearman–Brown split-half correction to the correlation. ^α^ Reliability was estimated using Cronbach’s α on accuracy

**Table 4:** Zero-order correlations among measures for Attempt 1

Measure	1	2	3	4	5	6	7	8	9	10	11	12
1. Antisaccade	–											
2. PVT	**–.41**	–										
3. SART	**–.37**	**.41**	–									
4. Stroop^2^	**.29**	**–.27**	**–.32**	–								
5. Flanker^2^	**.33**	**–.25**	**–.47**	**.45**	–							
6. Simon^2^	**.34**	**–.41**	**–.42**	**.49**	**.49**	–						
7. Choice RT	**–.42**	**.39**	**.36**	**–.45**	**–.29**	**–.52**	–					
8. Letter comp	**.22**	–.08	–.13	.16	**.25**	**.26**	**–.25**	–				
9. Digit comp	**.24**	**–.25**	**–.29**	.20	**.37**	**.45**	**–.27**	.**48**	–			
10. OSpan	**.27**	–.14	–.17	.21	**.34**	**.26**	**–.23**	.21	**.26**	–		
11. SymSpan	**.25**	–.07	–.12	**.28**	**.26**	**.28**	**–.22**	.08	**.24**	**.35**	–	
12. RSpan	**.31**	–.19	**–.34**	**.28**	**.40**	**.37**	**–.24**	.**26**	**.35**	**.56**	.40	–
13. Color CD	**.35**	**–.26**	**–.39**	**.30**	**.31**	**.28**	–.18	.06	**.23**	.20	.22	**.25**
14. Orient. CD	**.46**	**–.35**	**–.45**	**.34**	**.39**	**.38**	**–.29**	.14	**.30**	**.25**	.24	**.37**
15. Letter CD	**.42**	**–.35**	**–.48**	.20	**.37**	**.34**	**–.23**	.19	**.33**	**.28**	.18	**.34**
16. IFR	**.30**	**–.22**	**–.42**	**.30**	**.37**	**.28**	**–.25**	.16	**.24**	**.30**	**.24**	**.46**
17. Cued recall	**.34**	**–.31**	**–.36**	**.28**	**.36**	.16	**–.23**	.06	.08	.11	.11	**.22**
18. Recognition	**.33**	**–.32**	**–.40**	.19	**.30**	.21	**–.28**	–.04	.14	.13	.08	**.28**
19. Raven	**.35**	**–.23**	**–.36**	**.49**	**.45**	**.40**	**–.40**	**.22**	**.25**	**.30**	**.34**	**.41**
20. Num. series	**.24**	**–.12**	**–.30**	**.25**	**.40**	**.35**	**–.26**	**.22**	**.28**	**.37**	**.32**	**.37**
21. Letter sets	**.30**	**–.16**	**–.44**	**.30**	**.35**	**.27**	–.22	**.25**	.21	.19	.20	**.30**
22. Paper fold	**.30**	**–.17**	**–.36**	**.40**	**.50**	**.35**	**–.38**	.16	.20	.18	**.32**	**.27**
23. SAT	**.35**	**–.25**	**–.39**	**.38**	**.45**	**.37**	**–.40**	.16	**.29**	**.26**	**.32**	**.34**
24. TOT	**.32**	**–.30**	**–.41**	**.37**	**.40**	**.38**	**–.37**	.13	.20	**.29**	**.37**	**.33**

Measure	1	13	14	15	16	17	18	19	20	21	22	23
1. Antisaccade	–											
2. PVT	**–.41**											
3. SART	**–.37**											
4. Stroop^2^	**.29**											
5. Flanker^2^	**.33**											
6. Simon^2^	**.34**											
7. Choice RT	**–.42**											
8. Letter comp	**.22**											
9. Digit comp	**.24**											
10. OSpan	**.27**											
11. SymSpan	**.25**											
12. RSpan	**.31**											
13. Color CD	**.35**	–										
14. Orient. CD	**.46**	**.47**	–									
15. Letter CD	**.42**	**.46**	**.63**	–								
16. IFR	**.30**	**.25**	**.44**	**.31**	–							
17. Cued recall	**.34**	**.34**	**.40**	**.30**	**.52**	–						
18. Recognition	**.33**	**.28**	**.45**	**.36**	**.48**	**.50**	–					
19. Raven	**.35**	**.33**	**.50**	**.39**	**.40**	**.32**	**.29**	–				
20. Num. series	**.24**	**.24**	**.31**	**.30**	**.32**	.17	.18	**.40**	–			
21. Letter sets	**.30**	**.26**	**.42**	**.38**	**.34**	**.33**	**.29**	**.36**	**.42**	–		
22. Paper fold	**.30**	**.26**	**.42**	**.34**	**.35**	**.38**	**.38**	**.55**	**.36**	**.40**	–	
23. SAT	**.35**	**.27**	**.43**	**.32**	**.33**	**.39**	**.29**	**.53**	**.30**	**.29**	**.60**	–
24. TOT	**.32**	**.32**	**.38**	**.28**	**.29**	**.32**	**.29**	**.55**	**.25**	**.32**	**.58**	**.65**

*PVT* psychomotor vigilance task, *SART* Sustained Attention to Response Task, *RT* reaction time, *comp*. comparison, *OSpan* operation span, *SymSpan* symmetry span, *RSpan* reading span, *CD* change detection, *IFR* immediate free recall, *Num. series* number series, *SAT* spatial apperception test, *TOT* terrain orientation task. Bolded correlations are significant at *p* <.001

**Table 5:** Zero-order correlations among measures for Attempt 2

Measure	1	2	3	4	5	6	7	8	9	10	11	12
1. Antisaccade	–											
2. PVT	**–.55**	–										
3. SART	**–.54**	**.48**	–									
4. Stroop^2^	**.33**	–.17	**–.38**	–								
5. Flanker^2^	**.52**	**–.34**	**–.48**	**.53**	–							
6. Simon^2^	**.40**	**–.33**	**–.42**	**.52**	**.54**	–						
7. Choice RT	**–.44**	**.39**	**.34**	**–.37**	**–.46**	**–.47**	–					
8. Letter comp	.15	–.21	–.13	**.26**	.19	**.28**	–.17	–				
9. Digit comp	.09	–.14	–.13	.15	.21	**.40**	**–.28**	**.50**	–			
10. OSpan	.18	–.11	–.12	**.25**	**.33**	**.23**	**–.25**	.19	.19	–		
11. SymSpan	**.28**	**–.24**	**–.22**	**.37**	**.45**	**.34**	**–.44**	.15	.14	**.28**	–	
12. RSpan	**.32**	–.22	**–.30**	**.33**	**.37**	**.30**	**–.23**	.16	.16	**.56**	**.36**	–
13. Color CD	**.43**	**–.32**	**–.47**	**.24**	**.33**	**.22**	**–.15**	.16	.09	.16	.20	**.31**
14. Orient. CD	**.46**	**–.34**	**–.46**	**.27**	**.43**	**.26**	**–.30**	.13	.12	.**22**	**.34**	**.28**
15. Letter CD	**.48**	**–.39**	**–.46**	**.26**	**.38**	**.33**	**–.26**	.07	.08	.20	**.23**	**.32**
16. IFR	**.37**	**–.38**	**–.40**	**.33**	**.48**	**.27**	**–.32**	.22	.18	**.39**	**.29**	**.49**
17. Cued recall	**.34**	**–.25**	**–.33**	**.33**	**.42**	.21	**–.30**	.11	.00	**.26**	**.28**	**.38**
18. Recognition	**.50**	**–.39**	**–.45**	**.32**	**.42**	**.27**	**–.31**	.07	.04	.20	.18	**.38**
19. Raven	**.42**	**–.27**	**–.52**	**.44**	**.57**	**.35**	**–.38**	.12	.18	**.29**	**.40**	**.37**
20. Num. series	**.37**	**–.24**	**–.24**	**.35**	**.45**	**.37**	**–.29**	.15	**.25**	**.36**	**.35**	**.36**
21. Letter sets	**.40**	**–.27**	**–.39**	**.30**	**.48**	**.28**	**–.26**	.10	.09	**.26**	**.29**	**.34**
22. Paper fold	**.49**	**–.34**	**–.44**	**.46**	**.58**	**.32**	**–.36**	.11	.00	.19	**.41**	**.30**
23. SAT	**.48**	**–.36**	**–.42**	**.41**	**.53**	**.41**	**–.46**	.05	.14	**.32**	**.41**	**.35**
24. TOT	**.39**	**–.25**	**–.36**	**.40**	**.51**	**.30**	**–.39**	.11	.17	**.35**	**.36**	**.37**

Measure	1	13	14	15	16	17	18	19	20	21	22	23
1. Antisaccade	–											
2. PVT	**–.55**											
3. SART	**–.54**											
4. Stroop^2^	**.33**											
5. Flanker^2^	**.52**											
6. Simon^2^	**.40**											
7. Choice RT	**–.44**											
8. Letter comp	.15											
9. Digit comp	.09											
10. OSpan	.18											
11. SymSpan	**.28**											
12. RSpan	**.32**											
13. Color CD	**.43**	–										
14. Orient. CD	**.46**	**.55**	–									
15. Letter CD	**.48**	**.57**	**.61**	–								
16. IFR	**.37**	**.34**	**.47**	**.35**	–							
17. Cued recall	**.34**	**.29**	**.41**	**.34**	**.56**	–						
18. Recognition	**.50**	**.39**	**.41**	**.48**	**.55**	**.51**	–					
19. Raven	**.42**	**.44**	**.48**	**.39**	**.50**	**.40**	**.42**	–				
20. Num. series	**.37**	**.24**	**.34**	**.27**	**.40**	**.25**	**.31**	**.45**	–			
21. Letter sets	**.40**	**.32**	**.36**	**.40**	**.39**	**.28**	**.37**	**.49**	**.47**	–		
22. Paper fold	**.49**	**.41**	**.50**	**.39**	**.42**	**.42**	**.46**	**.64**	**.41**	**.52**	–	
23. SAT	**.48**	**.34**	**.43**	**.37**	**.38**	**.36**	**.35**	**.63**	**.40**	**.37**	**.65**	–
24. TOT	**.39**	**.38**	**.42**	**.30**	**.40**	**.32**	**.35**	**.58**	**.44**	**.45**	**.66**	**.68**

*PVT* psychomotor vigilance task, *SART* Sustained Attention to Response Task, *RT* reaction time, *comp*. comparison, *OSpan* operation span, *SymSpan* symmetry span, *RSpan* reading span, *CD* change detection, *IFR* immediate free recall, *Num. series* number series, *SAT* spatial apperception test, *TOT* terrain orientation task. Bolded correlations are significant at *p* <.001

Table 6 summarizes the test–retest reliability and practice effects. Most measures showed acceptable test–retest reliability. The exceptions were the PVT, letter comparison, digit comparison, symmetry span, and color change-detection tasks. Fourteen out of 24 measures did not show a significant practice effect. Whereas some tasks (Stroop^2^, Flanker^2^, letter sets, and terrain orientation) showed significant improvement on Attempt 2, a few tasks (PVT, orientation change detection, recognition, and Raven) showed significantly worse performance on Attempt 2.

**Table 6:** Test–retest reliability and practice effects for individual measures

	Test–retest reliability		Practice effect				
Measure	*r*	ICC	*t*	*df*	*p*	*d*	95% CI
Antisaccade	.59	.73	1.16	237	.249	0.05	[0.23, –0.12]
***Psychomotor vigilance***	***.52***	***.66***	***7.05***	***199***	** < *.001***	***0.56***	***[0.75, 0.37]***
SART	.67	.81	3.33	202	.001	0.16	[0.35, –0.02]
**Stroop**^**2**^	**.80**	**.89**	**10.22**	**230**	** <.001**	**0.41**	**[0.59, 0.23]**
**Flanker**^**2**^	**.73**	**.85**	**8.17**	**232**	** <.001**	**0.37**	**[0.55, 0.19]**
Simon^2^	.69	.82	2.48	225	.014	0.14	[0.32, –0.05]
Choice reaction time	.78	.89	1.13	216	.260	0.02	[0.21, –0.16]
Letter comparison	.34	.51	1.77	217	.079	0.10	[0.28, –0.08]
Digit comparison	.52	.69	1.95	216	.053	0.17	[0.36, –0.01]
**Operation span**	**.57**	**.76**	**3.86**	**222**	** <.001**	**0.27**	**[0.45, 0.08]**
Symmetry span	.50	.67	2.88	222	.004	0.21	[0.39, 0.03]
Reading span	.57	.72	–1.63	235	.105	–0.11	[0.07, –0.29]
Color change detection	.47	.64	–2.53	234	.012	–0.20	[–0.02, –0.37]
***Orientation change detection***	***.61***	***.75***	***–4.44***	***237***	** < *.001***	***–0.27***	***[–0.09, –0.45]***
Letter change detection	.59	.74	–2.09	238	.038	–0.13	[0.04, –0.31]
Immediate free recall	.68	.80	1.88	216	.061	0.09	[0.27, –0.09]
Cued recall	.73	.85	–1.46	229	.146	–0.03	[0.15, –0.21]
***Word recognition***	***.70***	***.83***	***–6.01***	***223***	** < *.001***	***–0.28***	***[–0.10, –0.46]***
***Raven***	***.71***	***.83***	***–8.62***	***238***	** < *.001***	***–0.45***	***[–0.27, –0.62]***
Number series	.67	.81	–1.80	239	.073	–0.11	[0.07, –0.29]
**Letter sets**	**.55**	**.71**	**5.63**	**215**	** <.001**	**0.31**	**[0.49, 0.12]**
Paper folding	.78	.88	1.28	234	.203	0.05	[0.23, –0.13]
Spatial apperception	.71	.86	3.41	236	.001	0.14	[0.32, –0.04]
**Terrain orientation**	**.74**	**.85**	**4.59**	**221**	** <.001**	**0.20**	**[0.38, 0.02]**

*r* Pearson’s correlation between the first and second attempt; *ICC* intraclass correlation coefficient. *SART* Sustained Attention to Response Task, *df* degrees of freedom, *d* effect size, *CI* confidence interval around effect size estimate. Bolded practice effects represent significantly better performance on Attempt 2, whereas bolded and italicized rows indicate significantly worse performance on Attempt 2

### Factor analysis

**Fig. 1:**
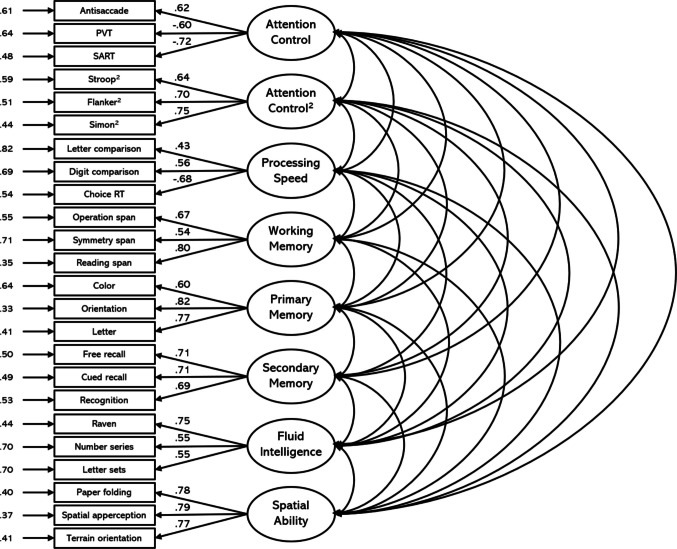
Confirmatory factor analysis for Attempt 1. *Note.* Boxes indicate manifest variables (individual task measures); ovals represent latent variables; numbers to the left of manifest variables indicate standardized residual variances; numbers on paths between latent and manifest variables represent standardized factor loadings. All factor loadings and interfactor correlations are significant at *p* <.001

We submitted the 24 measures to a confirmatory factor analysis with each measure loading onto a latent variable representing its hypothesized cognitive construct. From the outset we decided to split the “traditional” attention control tasks (antisaccade, PVT, and SART) and the “squared” attention control tasks onto separate factors, as these have been shown to load onto different latent factors in prior work (Burgoyne et al., 2023). The model fit the data well for the Attempt 1 data, χ^2^(224) = 370.13, CFI =.93, TLI =.92, RMSEA =.050 [.041,.060], SRMR =.05 (see Fig. 1). The latent correlations among the factors for Attempt 1 are listed in Table 7. For the most part, the pattern of latent correlations was consistent with prior work. There was a positive manifold in the data, such that individuals who performed highly on one ability tended to perform highly on measures of other abilities. Some notable correlations include the correlation between the Attention Control and Attention Control^2^ factors (*r* =.80), which suggests some distinctiveness, and the high correlation between the Attention Control^2^ and Processing Speed factors (*r* =.90). Both correlations warrant further discussion, and we will return to these points later.

**Table 7:** Latent variable correlations for Attempt 1

Latent factor	1	2	3	4	5	6	7
1. Attention Control	–						
2. Attention Control^2^	.80	–					
3. Processing Speed	.83	.90	–				
4. Working Memory	.56	.61	.62	–			
5. Primary Memory	.86	.63	.57	.53	–		
6. Secondary Memory	.76	.54	.47	.45	.67	–	
7. Fluid Intelligence	.75	.84	.75	.72	.78	.65	–
8. Spatial Ability	.66	.74	.64	.52	.59	.61	.84

All correlations are significant at *p* <.001

**Fig. 2:**
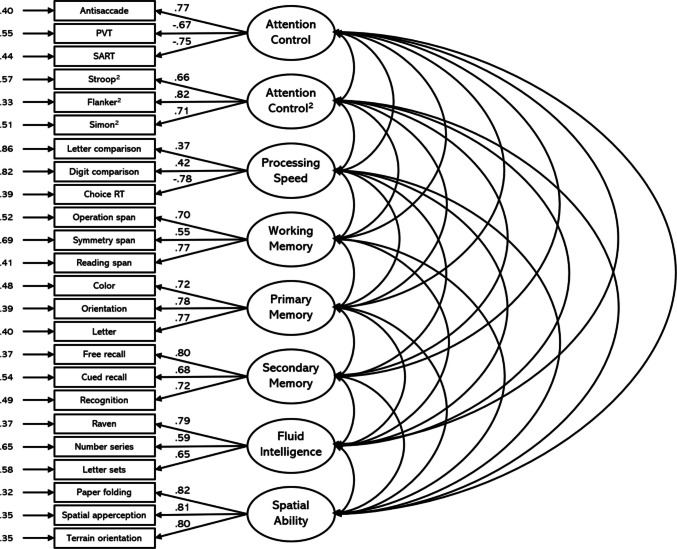
Confirmatory factor analysis for Attempt 2. *Note.* Boxes indicate manifest variables (individual task measures); ovals represent latent variables; numbers to the left of manifest variables indicate standardized residual variances; numbers on paths between latent and manifest variables represent standardized factor loadings. All factor loadings and interfactor correlations are significant at *p* <.001

The model also fit the data well for the Attempt 2 data, χ^2^(224) = 427.24, CFI =.92, TLI =.90, RMSEA =.060 [.052,.069], SRMR =.06 (see Fig. 2). The latent correlations among the factors for Attempt 2 are listed in Table 8. In general, the factor loadings and interfactor correlations were similar when the model was fit to the Attempt 2 data. Again, there was only a moderate correlation between the Attention Control and Attention Control^2^ factors (*r* =.74), indicating distinctiveness; however, the correlation between the Attention Control^2^ and Processing Speed factors dropped slightly (*r* =.82). Further, there was a large correlation between the Fluid Intelligence and Spatial Ability factors (*r* =.87).

**Table 8:** Latent variable correlations for Attempt 2

Latent factor	1	2	3	4	5	6	7
1. Attention Control	–						
2. Attention Control^2^	.74	–					
3. Processing Speed	.65	.82	–				
4. Working Memory	.49	.63	.55	–			
5. Primary Memory	.78	.57	.41	.51	–		
6. Secondary Memory	.71	.65	.54	.70	.67	–	
7. Fluid Intelligence	.74	.83	.61	.71	.71	.64	–
8. Spatial Ability	.68	.76	.60	.58	.64	.60	.87

All correlations are significant at *p* <.001

**Fig. 3:**
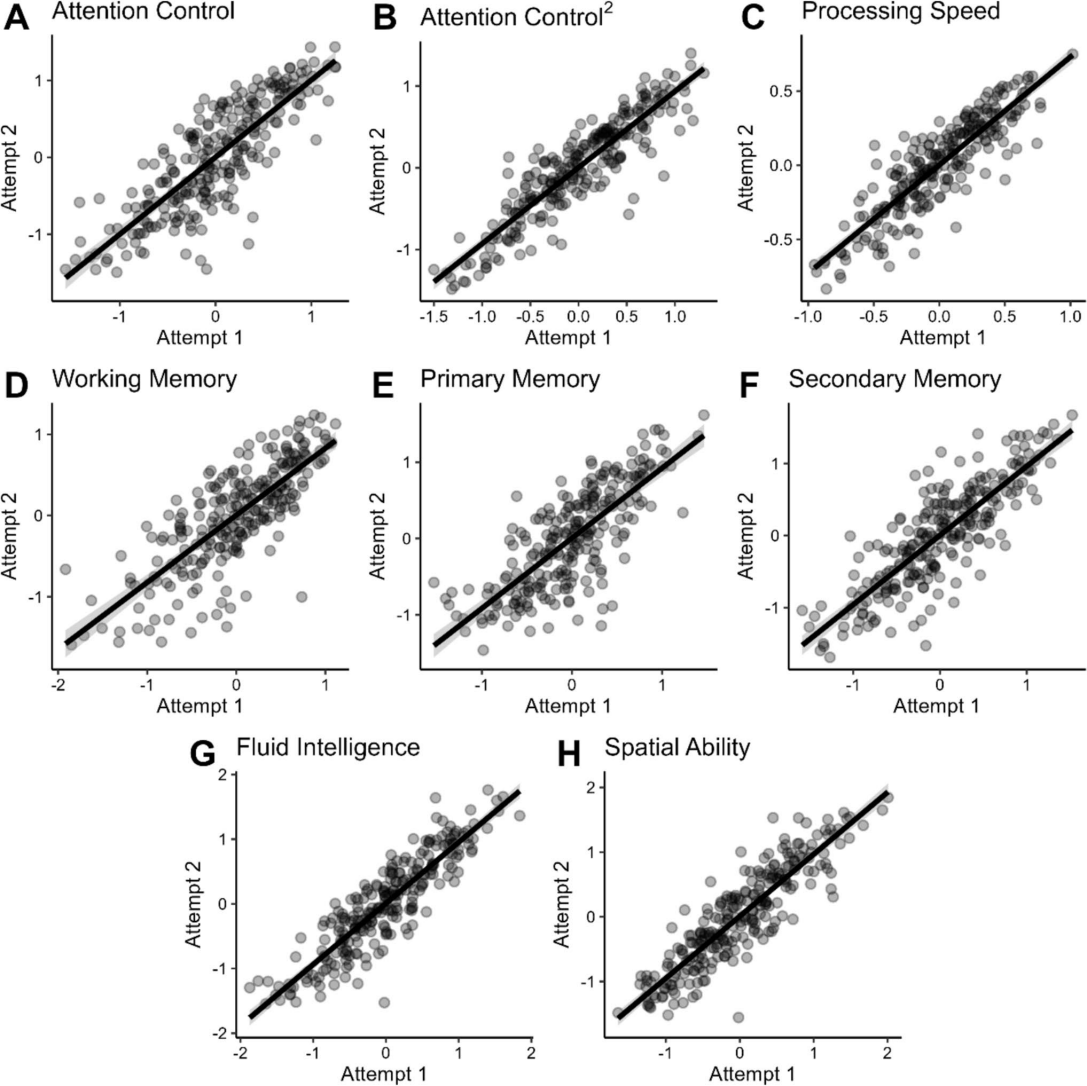
Scatterplots of factor scores for each participant across attempts. *Note*. Points on each plot represent an individual. Values on axes are factor scores extracted from the confirmatory factor analyses. The solid black line shows the line of best fit through the points to visualize the strong positive correlations

To estimate construct-level reliability, we extracted factor scores for each of the eight factors for Attempt 1 and Attempt 2 and correlated the factor scores across attempts using the *regression* method of the *lavPredict()* function in *lavaan*. Scatterplots of these correlations are shown in Fig. 3. Reliability was estimated by correlating the factor scores derived from the Attempt 1 model and the Attempt 2 model. Overall, the construct-level scores were highly reliable. That is, people who demonstrated relatively high or low performance on a set of representative measures tended to do so consistently across attempts. Reliability estimates for each factor score are listed in Table 9.

**Table 9:** Construct-level reliability estimates

Construct	*r*	ICC
Attention Control	.83	.89
Attention Control^2^	.90	.97
Processing Speed	.88	.93
Working Memory	.78	.88
Primary Memory	.77	.86
Secondary Memory	.84	.91
Fluid Intelligence	.89	.97
Spatial Ability	.89	.97

Next, we examined the extent to which the factor structure changed from Attempt 1 to Attempt 2 with tests of measurement invariance. First, we specified a model in which the Attempt 1 and Attempt 2 data were specified to come from two groups, with each parameter freely estimated in each group. This is equivalent to specifying the model separately for each attempt, as we did above. Then, we specified a second model in which the factor loadings were forced to be equal across attempts. Doing so did not worsen the model fit compared to the free estimation in each attempt, Δχ^2^(16) = 11.04, *p* =.81. A Bayes factor (BF) comparison yielded strong evidence in favor of no difference in factor loadings (BF > 100,000). Therefore, the factor loadings were roughly equal when the model was fit to each attempt. Then, we specified a third model in which both the factor loadings and the interfactor correlations were forced to be equal and again compared it back to the freely estimated model. Doing so did not worsen the model fit, Δχ^2^(28) = 19.74, *p* =.87, BF > 100,000. Therefore, the interfactor correlations were about equal across attempts as well. Thus, there was invariance in the factor structure across repeated attempts of the cognitive battery.

### Jingle-jangle fallacy tests

As mentioned earlier, there are many theoretical questions of interest that may be addressed with these data. But here we will focus on a few instances in which convergent and discriminant validity need to be demonstrated. The first question considers whether the “traditional” attention control measures (i.e., antisaccade, PVT, and SART) and newer “squared” attention control measures (i.e., Stroop^2^, Flanker^2^, and Simon^2^) measure qualitatively different constructs (*jingle* fallacy). Indeed, this seemed to be the case, as in both Attempt 1 and Attempt 2, the correlations between the two factors were significant, but indicated distinctiveness. As a specific test, we re-specified two models, one allowing all six measures to load onto a single factor, then compared that model back to a model in which the “traditional” and “squared” factors loaded onto separate factors. The models were fit separately to the Attempt 1 and Attempt 2 data. For Attempt 1, separating the measures onto two factors significantly improved the model fit, Δχ^2^(1) = 15.18, *p* <.001. A BIC comparison yielded strong evidence in favor of separating the factors (BF = 124.05). Loading the measures onto two factors also significantly improved the fit for the Attempt 2 data, Δχ^2^(1) = 40.35, *p* <.001, BF > 100,000. Thus, the “traditional” measures of attention control and the “squared” measures of attention control seem to measure some overlapping but also some distinct underlying sources of variance.

The next issue is whether the high correlation between the Attention Control and Processing Speed factors represents an issue of discriminant validity. That is, we may be committing a *jangle* fallacy by assuming their measures are tapping into two different constructs. To assess this, we specified a model in which antisaccade, PVT, SART, letter comparison, digit comparison, and choice reaction time tasks all loaded onto a single factor and fit it to the Attempt 1 and Attempt 2 data. Then we compared the model fits to models in which the attention and speed tasks loaded onto separate factors. For both Attempt 1, Δχ^2^(1) = 5.41, *p* =.02, and Attempt 2, Δχ^2^(1) = 18.39, *p* =.03, the two-factor model fit the data significantly better. The BF comparison was equivocal for Attempt 1 (BF in favor of two-factor model = 0.93), but strongly favored the two-factor solution for Attempt 2 (BF = 625.33). We repeated this analysis with the “squared” attention tasks. For Attempt 1, there was a nonsignificant difference between the one- and two-factor model fits, Δχ^2^(1) = 2.68, *p* =.10, indicating the measures could adequately be explained by a single underlying construct. A BF comparison corroborated this finding (BF in favor of the single-factor model = 4.11) However, for Attempt 2, there was a significant improvement to model fit when specifying two separate factors, Δχ^2^(1) = 4.90, *p* =.03. The BF comparison here was equivocal (BF in favor of single-factor model = 1.35).

As a third test of a potential *jangle* fallacy, we examined the Fluid Intelligence and Spatial Ability factors. Their correlation was particularly high in the Attempt 2 data. Therefore, we specified a model in which all six Raven, number series, letter sets, terrain orientation, spatial apperception, and paper folding tasks were allowed to load onto a single factor, and compared that back to the original seven-factor model. For Attempt 1, the single-factor model fit acceptably, and loading the measures onto separate factors fit the data significantly better than a single-factor model, Δχ^2^(1) = 12.68, *p* <.001. A BIC comparison strongly favored the two-factor solution (BF = 35.78). For Attempt 2, loading the measures onto separate factors also fit significantly better, Δχ^2^(1) = 7.96, *p* =.005, BF = 3.39. Therefore, despite the high correlation between the Fluid Intelligence and Spatial Ability factors, there was still evidence in favor of keeping them specified as separate factors.

### Latent state-trait analysis

**Fig. 4:**
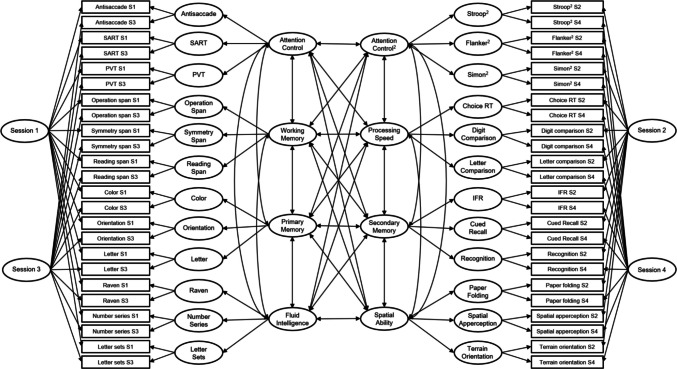
Latent state-trait model. *Note.* Single-headed arrows represent factor loadings; double-headed arrows represent latent factor correlations. Boxes indicate manifest variables (individual task measures); ovals represent latent variables; numbers to the left of manifest variables indicate standardized residual variances; numbers on paths between latent and manifest variables represent standardized factor loadings. Refer to Table 10 for parameter estimates of the model

A final factor analytic model was specified to partition variance in each measure into trait-level, task-specific, state-specific, and error variance. The model was applied to all 48 measures and is shown in Fig. 4. The model comprised eight trait factors, four state-specific factors, and 24 task-specific factors (see Analysis section for additional details on model estimation). The resulting model fit the data acceptably well, χ^2^(1,028) = 1,585.31, CFI =.92, TLI =.91, RMSEA =.046 [.042,.051], SRMR =.08. All parameter estimates that were not forced to be zero in the model are listed in Table 10. Path tracing of the model-estimated parameters was used to partition variance, and the results are summarized in Table 11. The largest portion of variance was attributable to trait-level factors (average =.46), the second-largest to residual/error variance (average =.32); the third-largest was task-specific variance (average =.18); and very small proportions were due to state-specific factors (average =.03). Table 12 lists the correlations among the trait factors. These were largely similar to the correlations observed in the confirmatory factor models.

**Table 10:** Parameter estimates from latent state-trait model

		Task factor loadings		State factor loadings		Trait factor loading		Error/residual variance	
Measure	Session	Est	*SE*	Est	*SE*	Est	*SE*	Est	*SE*
Antisaccade	1	.73	.03	.20	.05	.85	.04	.42	.04
	3	.78	.03	.29	.07			.30	.05
SART	1	.81	.03	–.27	.06	–.84	.04	.29	.05
	3	.81	.03	–.30	.07			.26	.05
PVT	1	.74	.04	.19	.05	–.76	.05	.26	.05
	3	.74	.04	–.36	.07			.37	.06
Operation span	1	.78	.03	.13	.04	.88	.04	.39	.05
	3	.80	.03	.00	.07			.36	.05
Symmetry span	1	.71	.04	.12	.05	.73	.06	.50	.05
	3	.73	.03	.03	.06			.47	.06
Reading span	1	.77	.03	.02	.05	1.00	.04	.40	.05
	3	.74	.03	.16	.06			.43	.06
Color	1	.68	.04	.25	.06	.84	.05	.48	.05
	3	.72	.04	.35	.06			.36	.06
Orientation	1	.81	.03	.29	.05	.98	.03	.30	.05
	3	.77	.03	.24	.06			.36	.04
Letter	1	.76	.03	.29	.07	.92	.03	.34	.05
	3	.74	.03	.37	.06			.31	.05
Raven	1	.84	.02	.13	.04	.89	.03	.28	.04
	3	.86	.02	.17	.05			.24	.04
Number series	1	.81	.03	.06	.05	.70	.03	.35	.04
	3	.84	.03	.06	.05			.30	.05
Letter sets	1	.73	.03	.12	.05	.79	.05	.46	.05
	3	.76	.03	.16	.06			.40	.05
Stroop^2^	2	.89	.02	.14	.07	.74	.05	.19	.04
	4	.88	.02	.28	.06			.16	.04
Flanker^2^	2	.87	.02	.16	.09	.82	.04	.22	.04
	4	.85	.02	.32	.06			.18	.04
Simon^2^	2	.85	.02	.18	.08	.89	.03	.19	.04
	4	.85	.02	.35	.07			.16	.04
Choice RT	2	.90	.02	–.02	.03	–.56	.07	.19	.04
	4	.90	.02	–.04	.05			.20	.04
Digit comparison	2	.73	.04	.09	.05	1.00	.00	.47	.05
	4	.70	.05	.17	.09			.48	.05
Letter comparison	2	.59	.04	.08	.05	1.00	.00	.64	.03
	4	.59	.04	.15	.09			.63	.05
IFR	2	.80	.03	.01	.03	.89	.04	.35	.04
	4	.85	.03	.01	.07			.27	.04
Cued recall	2	.85	.02	.01	.03	.76	.04	.29	.04
	4	.87	.02	.01	.06			.25	.04
Recognition	2	.84	.02	.05	.03	.84	.04	.29	.04
	4	.84	.02	.09	.07			.29	.04
Paper folding	2	.88	.02	.08	.05	.88	.03	.22	.03
	4	.89	.02	.16	.06			.18	.03
SAT	2	.81	.02	.07	.04	.87	.03	.35	.04
	4	.88	.02	.14	.06			.21	.04
TOT	2	.84	.02	.02	.03	.90	.03	.29	.04
	4	.89	.02	.04	.06			.21	.04

*Est*. standardized estimate, *SE* standard error of estimate. All other parameters in the model were set to equal 0. Trait factor loadings listed are the loadings of that task’s factor onto the trait factor

**Table 11:** Variance partitioning analysis using latent state-trait model

Factor	Task		Session	Trait-level		Task-specific	State-specific	Reliability
Attention	Antisaccade		1	.39		.15	.04	.58
Control			3	.44		.17	.08	.70
	SART		1	.46		.20	.05	.71
			3	.46		.20	.09	.74
	PVT		1	.32		.23	.07	.63
			3	.32		.23	.13	.68
*Factor average*				*.40*		*.20*	*.08*	*.67*
Working	Operation span		1	.50		.14	.00	.64
Memory			3	.49		.14	.00	.63
	Symmetry span		1	.27		.23	.01	.50
			3	.28		.24	.01	.53
	Reading span		1	.59		.00	.02	.60
			3	.54		.00	.03	.57
*Factor average*				*.45*		*.12*	*.01*	*.58*
Primary	Color		1	.32		.13	.06	.52
Memory			3	.36		.15	.13	.64
	Orientation		1	.63		.03	.04	.70
			3	.56		.03	.06	.64
	Letter		1	.49		.09	.08	.66
			3	.47		.08	.14	.69
*Factor average*				*.47*		*.10*	*.09*	*.64*
Fluid	Raven		1	.55		.15	.02	.72
Intelligence			3	.57		.16	.03	.76
	Number series		1	.32		.33	.00	.65
			3	.35		.36	.00	.71
	Letter sets		1	.33		.20	.01	.54
			3	.36		.22	.03	.60
*Factor average*				*.41*		*.24*	*.02*	*.66*
Attention		Stroop^2^	2		.43	.36	.02	.81
Control^2^			4		.42	.35	.08	.84
		Flanker^2^	2		.51	.24	.03	.78
			4		.49	.23	.10	.82
		Simon^2^	2		.62	.16	.03	.81
			4		.57	.15	.12	.84
*Factor average*					*.51*	*.25*	*.38*	*.82*
Processing		Choice RT	2		.26	.55	.00	.81
Speed			4		.25	.54	.00	.80
		Digit comp	2		.53	.00	.01	.53
			4		.50	.00	.01	.49
		Letter comp	2		.35	.00	.01	.46
			4		.35	.00	.02	.37
*Factor average*					*.37*	*.18*	*.01*	*.58*
Secondary		IFR	2		.52	.13	.00	.65
Memory			4		.58	.15	.00	.73
		Cued recall	2		.42	.30	.00	.71
			4		.44	.31	.00	.75
		Recognition	2		.50	.21	.00	.71
			4		.50	.20	.01	.71
*Factor average*					*.49*	*.22*	*.00*	*.71*
Spatial		Paper folding	2		.60	.17	.01	.78
Ability			4		.62	.18	.03	.82
		SAT	2		.49	.16	.00	.66
			4		.58	.19	.02	.79
		TOT	2		.57	.14	.00	.71
			4		.63	.16	.00	.79
*Factor average*					*.58*	*.17*	*.01*	*.76*
*Grand average*					*.46*	*.18*	*.03*	*.68*

*SART* Sustained Attention to Response Task, *PVT* psychomotor vigilance task, *IFR* immediate free recall, *SAT* spatial apperception test, *TOT* terrain orientation task. *Reliability* 1 – error variance

**Table 12:** Correlations among latent trait variables from latent state-trait model

Trait factor	1	2	3	4	5	6	7
1. Attention Control	–						
2. Attention Control^2^	.77	–					
3. Processing Speed	.53	.75	–				
4. Working Memory	.56	.68	.52	–			
5. Primary Memory	.80	.55	.44	.54	–		
6. Secondary Memory	.78	.47	.28	.60	.73	–	
7. Fluid Intelligence	.77	.75	.50	.71	.76	.73	–
8. Spatial Ability	.73	.67	.36	.57	.67	.62	.92

All correlations are significant at *p* <.001

### Drift–diffusion modeling of decision-making tasks

**Fig. 5:**
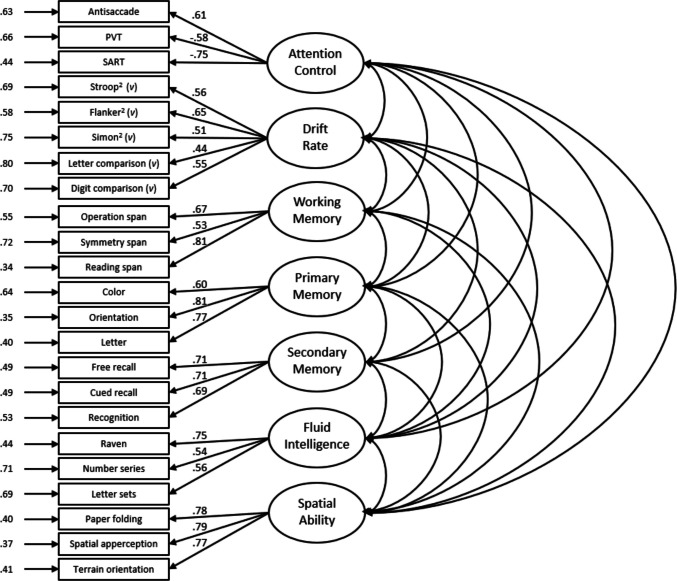
Confirmatory factor analysis using drift-rate parameters in Attempt 1. *Note.* Boxes indicate manifest variables (individual task measures); ovals represent latent variables; numbers to the left of manifest variables indicate standardized residual variances; numbers on paths between latent and manifest variables represent standardized factor loadings. All factor loadings and interfactor correlations are significant at *p* <.001

**Fig. 6:**
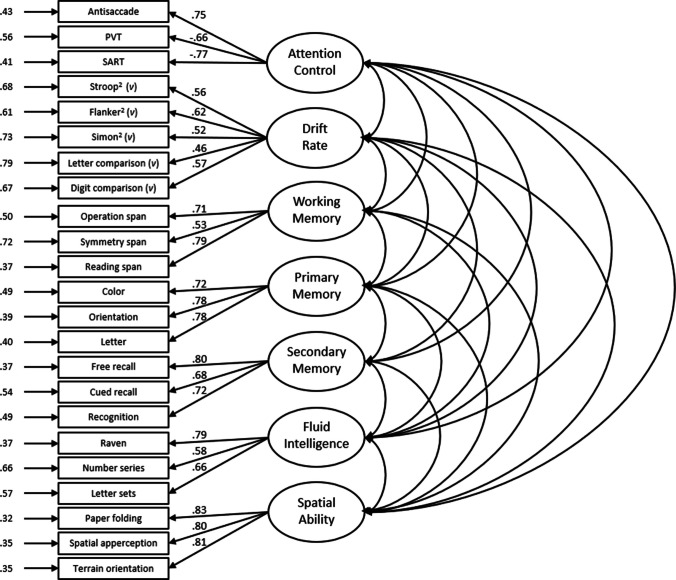
Confirmatory factor analysis using drift-rate parameters in Attempt 2. *Note.* Boxes indicate manifest variables (individual task measures); ovals represent latent variables; numbers to the left of manifest variables indicate standardized residual variances; numbers on paths between latent and manifest variables represent standardized factor loadings. All factor loadings and interfactor correlations are significant at *p* <.001

Given the high correlations between the “squared” attention control measures and the processing speed tasks, it is worth considering whether alternative measurement procedures would yield different results. For example, it has been demonstrated that re-scoring typical measures of executive functioning using drift–diffusion parameters (namely, drift rate) yields convergence upon a single common factor which correlates highly with working memory and fluid intelligence (Löffler et al., 2024).^4^ To address this question, we first submitted the RT and accuracy data from the Flanker^2^, Stroop^2^, and Simon^2^_,_ letter comparison, and digit comparison tasks to the EZ diffusion model (Barth, 2025; Wagenmakers et al., 2007).^5^ From these models we used the drift-rate parameter (*v*). Descriptive statistics for these parameters, including reliability, and correlations between all other measures are listed in the Appendix. We specified a confirmatory factor analysis in which drift rates from all five tasks loaded onto a factor. All other factors were specified the same as in Fig. 1. The model on Attempt 1 data fit well, χ^2^(209) = 332.15, CFI =.93, TLI =.92, RMSEA =.048 [.038,.058], SRMR =.05, and the drift rate factor correlated significantly with all other factors (see Fig. 5 and Table 13). This was also true for the Attempt 2 data, χ^2^(209) = 367.13, CFI =.93, TLI =.92, RMSEA =.055 [.046,.064], SRMR =.06 (see Fig. 6 and Table 14). These models show that the drift-rate parameters from these two-alternative forced-choice tasks were also reliable, correlated moderately with each other, and loaded onto a factor which was correlated with the other cognitive factors, replicating Löffler et al. (2024) and other work using drift–diffusion modeling parameters at the latent level (e.g., Schmiedek et al., 2007).

**Table 13:** Latent variable correlations including drift rate for Attempt 1

Latent factor	1	2	3	4	5	6	7
1. Attention Control	–						
2. Drift Rate	.77	–					
3. Working Memory	.56	.50	–				
4. Primary Memory	.86	.68	.53	–			
5. Secondary Memory	.76	.66	.46	.67	–		
6. Fluid Intelligence	.75	.77	.71	.78	.66	–	
7. Spatial Ability	.66	.67	.51	.59	.61	.84	–

All correlations are significant at *p* <.001

**Table 14:** Latent variable correlations including drift rate for Attempt 2

Latent factor	1	2	3	4	5	6	7
1. Attention Control	–						
2. Drift Rate	.73	–					
3. Working Memory	.49	.62	–				
4. Primary Memory	.78	.66	.50	–			
5. Secondary Memory	.71	.75	.70	.67	–		
6. Fluid Intelligence	.75	.82	.69	.71	.74	–	
7. Spatial Ability	.68	.65	.56	.64	.64	.91	–

All correlations are significant at *p* <.001

## Discussion

The present study evaluated the psychometric properties of a battery of 24 cognitive tests selected to measure the constructs of attention control, processing speed, working memory, primary memory, secondary memory, fluid intelligence, and spatial ability. This project was borne out of recent debates regarding the psychometric coherence and theoretical utility of attention control as a cognitive construct (Draheim et al., 2019, 2021, 2024; Enkavi et al., 2019; Hedge et al., 2018; Löffler et al., 2024; Mashburn et al., 2024; Rey-Mermet et al., 2018, 2019; Rey-Mermet, 2024; Robison et al., 2024a, 2024b; Rouder & Haaf, 2019; Rouder et al., 2023; Tsukahara et al., 2025; Unsworth et al., 2021, 2024; Unsworth & Miller, 2024; von Bastian et al., 2020). We administered the battery to participants twice, allowing for an assessment of test–retest reliability, the magnitude of practice effects, the factor structure of the constructs, the stability of that factor structure across two testing occasions, and the portions of variance in each measure attributable to task-specific, state-specific, and trait-level factors. Subsequently, we were able to test more theoretically meaningful questions about the psychometrics of attention control more specifically.

Most measures showed acceptable intrasession reliability on the first testing occasion (Attempt 1), with the only exception being the symmetry span task, which had a reliability estimate (.68) just below our cutoff for acceptability (>.70). On the second testing occasion, all measures had acceptable reliability estimates. Across testing occasions, most measures had acceptable test–retest reliability. The exceptions were the PVT (.66), the letter comparison task (.51), digit comparison (.69), symmetry span (.67) and color change detection (.64). Further, most measures showed minimal practice effects. The largest positive effects (Attempt 2 > Attempt 1) were observed in the Stroop^2^ (*d* = 0.41) and Flanker^2^ (*d* = 0.37) tasks, with significant practice effects also observed for the letter sets (*d* = 0.31) and terrain orientation tasks (*d* = 0.20). Some tasks showed significant *negative* practice effects. The PVT (*d* = 0.56),^6^ orientation change detection (*d* = − 0.27), word recognition (*d* = − 0.28), and Raven matrices (*d* = − 0.45) all demonstrated significantly lower performance on the second testing occasion than the first.

### Addressing the attention control debate

The assessment of psychometric properties discussed above was rather atheoretical. But as mentioned in the Introduction, a primary impetus for conducting this study was recent debate specifically regarding the construct validity of attention control. There is still active debate regarding what attention tasks and putative measures of related constructs like executive functioning (or executive control) actually measure. For example, some have argued that there is nothing “common” to measures of attention control, and therefore it is not a useful construct in theory (see Rey-Mermet et al., 2018, 2019; Rey-Mermet, 2024). On the other side of the debate are those who argue that, indeed, attention control can be measured reliably and a latent variable for it can be specified reliably, and it correlates with similar (and less controversial) constructs like working memory capacity and fluid intelligence (Mashburn et al., 2024; Robison et al., 2024a, 2024b; Unsworth et al., 2021, 2024). Further, some have argued that the common variance among measures of executive functioning measures is isomorphic with processing speed (Löffler et al., 2024). It is worth noting that most (although not all) of this argument centers on the use of conflict resolution tasks, like Stroop, Simon, and flanker tasks. However, Tsukahara et al. (2025) have recently argued that attention control is more than just conflict resolution, and that attention control is related to but distinct from processing speed. Amid this debate have been empirical reports on attempts to improve the psychometrics of attention control tasks either by adjusting the measures extracted from them or by designing new tasks that include aspects of stimulus–stimulus and stimulus–response conflict resolution, but critically do not use an RT difference score as the dependent measure (Burgoyne et al., 2023; Draheim et al., 2021, 2024; Unsworth & Miller, 2024). This is an important and oft-debated issue, and we therefore decided to use the present study as a way to bring more data to bear on these important theoretical and measurement questions.

Therefore, in the next set of analyses, we examined the latent structure of the abilities using confirmatory factor analysis. For the most part, the analyses confirmed our hypothesized structure. That is, a model loading the 24 tasks onto eight factors fit the data well for both the first and second attempts. Further, when factor scores were extracted from the models fit to the Attempt 1 and Attempt 2 data, robust correlations were found between the Attempt 1 and Attempt 2 factor scores. Therefore, construct-level estimates were highly reliable within individuals. But several findings from the factor analysis were notable and worth further discussion. First, the correlation between the Attention Control and Attention Control^2^ factors was robust, but did not suggest unity. For both attempts, loading the “traditional” and “squared” tasks onto separate factors fit the data significantly better. Thus, it would be a *jingle* fallacy to use the term “attention control” interchangeably to describe those two factors.

This result can be interpreted in one of two ways. First, it could be that the squared attention tasks and the traditional attention tasks measure qualitatively different abilities. Second, it could also be the case that because the tasks were given on different days, covariance among the measures also contains any temporally specific sources of variance present on that specific day of testing (e.g., fatigue, stress, motivation). However, in the first study that Burgoyne et al. (2023) report, there is a similar correlation (*r* =.80) between a factor formed by the squared tasks and a factor formed by “other” attention measures (antisaccade, selective visual arrays/change detection, and the SART task). Importantly, in this study, all six of the tasks were completed within the same session. In the second study reported by Burgoyne et al. (2023), the correlation was largely the same (*r* =.81) when the tasks were administered in different sessions. Further, our latent state-trait analyses indicated that very little variance in the measures could be attributed to state-specific factors. Therefore, the former explanation seems more likely: the squared tasks and “other” tasks measure some shared but also some distinct sources of interindividual variance.

The second issue of validity concerned discriminability between attention control and processing speed. On both attempts, there was a slightly higher correlation between the Processing Speed and Attention Control^2^ factors than there was between the Attention Control and Attention Control^2^ factors. In fact, a more parsimonious model could be constructed by allowing the three “squared” attention tasks (Flanker^2^, Stroop^2^, and Simon^2^) and the processing speed tasks onto a common factor for Attempt 1. However, for Attempt 2, there was evidence for separating them into two factors. Thus, the evidence for a *jangle* fallacy was equivocal here, and more data will be needed to assess the covariance between the squared tasks and typical measures of processing speed. Worth noting is the relatively low reliability of the letter comparison and digit comparison tasks. Further, the correlations between choice RT and the two comparison tasks (letter and digit) were lower than would be expected from measures of a common construct. For that reason, correlations will vary more widely from sample to sample. Therefore, extended and perhaps more varied measures of processing speed may need to be used in future research. Finally, in neither attempt was there evidence that Fluid Intelligence and Spatial Ability were isomorphic, despite their high correlation.

To summarize, we found that both the squared and more “traditional” measures of attention control were reliable, were intercorrelated, formed latent factors, and correlated with putatively related constructs like working memory capacity and fluid intelligence. Therefore, we would disagree with the notion that attention control should be abandoned as a construct. We acknowledge that we specifically did not, by design, include more traditional conflict resolution tasks that use an RT difference score as a dependent variable. It certainly seems possible that individual measures like the Stroop effect, Simon effect, and flanker effect are, at best, task-specific phenomena or, at worst, inconsistent and unreliable individual differences. Even so, we do not believe this observation necessitates the abandonment of attention control as a cognitive construct. Finally, we observed higher correlations between a factor formed by the “squared” attention control tasks and measures of processing speed than have been seen in prior work (Burgoyne et al., 2023), and those correlations were larger than those we observed between the “traditional” attention control factor and processing speed. Therefore, future work evaluating the psychometrics of these measures, especially their discriminant validity, seems warranted.

### State-, trait-, and task-specific variance

We found evidence for trait-general variability in performance in addition to some task-specific variance using latent state-trait modeling of the data. Very little variance in the measures was due to state-specific factors. Of the explainable variance in the 48 cognitive measures, about 69% was due to trait-level factors, about 26% to task-specific factors, and only about 4% to state-specific factors.^7^ Correlations among the trait-level factors were consistent with the confirmatory factor analyses. Thus, the largest proportion of systematic variance in the measures under investigation here was stable individual differences in latent abilities, the next-largest proportion being task factors (e.g., strategies, mnemonics, response mappings), and only very little due to factors unique to each measurement occasion (e.g., week-to-week variation in stress, fatigue, motivation).

### Limitations

Several methodological and analytical limitations of the current work are worth consideration. First, we are limited in our ability to generalize the findings outside the demographic range of our sample. Our participants were all students at a comprehensive state university in the United States. However, we would note that our sample was highly diverse and that the university from which the participants were sampled has a nationally representative population, being the sixth most diverse university in the United States, according to *U.S. News and World Report*, and a Hispanic and Asian American, Native American, and Pacific Islander-serving institution (University of Texas at Arlington, n.d.). That said, our findings may not generalize to populations in different age strata (e.g., children, older adults) or those with clinically significant cognitive impairments.

Second, some of our cognitive factors were formed by measures extracted from tasks with considerable shared method variance. For example, the Working Memory factor was specified with three complex span tasks; the Primary Memory factor was specified with three change-detection tasks; and the Secondary Memory factor was specified with three verbal list-learning paradigms. Therefore, the shared variance among those measures, represented by their common latent construct, will contain a blend of a true underlying cognitive construct but also any method-specific variance, such as encoding strategies or mnemonics.

Third, although it is possible to argue from the model comparisons that the “squared” tasks, which were designed to measure attention control (Burgoyne et al., 2023), measure nothing different from simpler perceptual discrimination tasks, any differentiation was complicated by shared method variance. Specifying separate factors for the “squared” attention tasks and the two comparison tasks would be uninformative because any differences between the factors could be due to heavy method overlap and not necessarily due to differences in the common/unique cognitive processes involved. Regardless, in future work, it will be worthwhile to additionally demonstrate convergence or distinctions between drift-rate parameters taken from “elementary” cognitive tasks, which make relatively few demands on conflict resolution, for example by stripping the conflict out of the “squared” tasks to see to what extent performance on their conflict-ridden versions is qualitatively different from the conflict-free versions.^8^

Fourth, our attention control tasks could be criticized because they did not have a “control” condition from which variance due to other factors, such as processing speed, could be partialed out (e.g., by subtracting accuracy on antisaccade trials from accuracy on prosaccade trials, congruent trials from incongruent trials). Therefore, the measures will not be as process-pure as we might hope. The particularly high correlations between the Attention Control and Attention Control^2^ factors with the Processing Speed factor indicate that perhaps much (or even most) of the systematic variance in attention control is due to individual differences in how quickly people can process information. Some recent work has directly addressed this notion. For example, Tsukahara et al. (2025) examined relations among inspection time measures of processing speed and found more evidence that processing speed, measured in various ways including inspection time, comparison tasks, and reaction time tasks, all had moderate to large correlations (~.60–.76) with their attention control factor. As mentioned earlier, studies like this will need to continue to determine whether and to what extent individual differences in attention control and processing speed differ from each other.

Finally, the effects of practice as measured here may have been limited by the relatively brief exposures to each task. For practice effects to appear, people may need more than a single previous exposure to see a benefit (Logan, 1988). Our goal here was not to allow participants to reach proficiency or automaticity, but to see whether in instances where people have previously encountered a test, for example in a clinic or laboratory, this poses a threat to its measurement properties. That said, it is possible that with more and repeated exposures, we may indeed have observed larger practice effects and potentially a shift in the factor structure.

## Conclusions

Overall, the present analyses indicate that a wide array of cognitive measures show acceptable and in some cases excellent psychometric properties. The measures tended to show high intrasession and test–retest reliability, low retesting/practice effects, and both convergent and discriminant validity. Novel to the present study was the finding of measurement invariance across testing occasions. That is, across multiple iterations of the same testing battery, the covariance structure among the tests remained largely unchanged. Importantly, the construct-level estimates exhibited high test–retest reliability for all eight factors in our latent variable model. This indicates that cognitive abilities can be measured reliably, that different abilities are correlated yet distinct, and that they demonstrate a consistent factor structure even across repeated testing. Despite these properties, we identified both an instance where a common term—“attention control”—has been used to label tasks which tap into clearly distinct variance, and an instance where some attention tasks were best modeled by loading onto a factor with measures of processing speed, particularly when employing parameters taken from drift–diffusion modeling. Therefore, more work is needed to assess the construct validity and discriminant validity of these measures.

## Data Availability

This project and all its associated data are hosted on the Open Science Framework (https://osf.io/j4tzh/). The analysis script for the present study is included as a component within that larger project (https://osf.io/v3adp/). Task materials are available from the authors upon request. Some tasks were developed using custom written Python scripts, others with custom written C# scripts.

## Footnotes

1 Participants were allowed to mark more than one racial/ethnic category.

2 Recently, Robison, Celaya et al., (2024a, 2024b) delivered a similar battery of 12 tasks measuring four abilities (primary memory, secondary memory, attention control, fluid intelligence) to about 200 participants each under conditions in which they fixed the sequence by grouping tasks by construct, fixed the sequence but interleaved the tasks by construct, or generated a random task sequence for each participant. Doing so did not systematically affect the zero-order correlations, factor loadings, or interfactor correlations. Therefore, it does not seem to carry a cost to delivering tasks in a fixed sequence versus a random sequence to each participant.

3 While it has been suggested that participants in the complex span tasks be screened for poor performance on the processing portion of the task (Conway et al., 2005), recent research has demonstrated that this criterion disproportionately removes low-performing participants, and therefore may not have the intended effect (Richmond et al., 2021). Therefore, we opted not to use any exclusion criteria for the complex span tasks beyond the outlier detection mentioned above.

4 We appreciate an anonymous reviewer suggesting this additional analysis.

5 We did not use the Choice RT data here because the task had four options, and therefore does not meet the same assumptions as these other five tasks.

6 The positive effect size here means reaction times increased, so performance decreased.

7 Note: these values are proportions of non-error variance, not raw proportions of variance.

8 Although we could have focused an analysis here on the no-conflict trials, analyzing only 25% of the trials in a 90-s tasks would have left us with very few trials for estimation.

## Declarations

### Open Practices Statement

The data presented here were part of a larger project. This project and all its associated data are hosted on the Open Science Framework (https://osf.io/j4tzh/). The analysis script for the present study is included as a component within that larger project (https://osf.io/v3adp/). Task materials are available from the authors upon request. Some tasks were developed using custom written Python scripts, others with custom written C# scripts. This study was not preregistered.
